# Sweat rate analysis of ivacaftor potentiation of CFTR in non-CF adults

**DOI:** 10.1038/s41598-018-34308-8

**Published:** 2018-11-02

**Authors:** Jeeyeon Kim, Miesha Farahmand, Colleen Dunn, Carlos E. Milla, Rina I. Horii, Ewart A. C. Thomas, Richard B. Moss, Jeffrey J. Wine

**Affiliations:** 1 Cystic Fibrosis Research Laboratory, Stanford University, Stanford, CA 94305 USA; 2 Department of Pediatrics, Stanford University School of Medicine, Stanford, CA 94305 USA; 3 Department of Psychology, Stanford University, Stanford, CA 94305 USA

## Abstract

To determine if ivacaftor (Kalydeco) influences non-CF human CFTR function *in vivo*, we measured CFTR-dependent (C-sweat) and CFTR-independent (M-sweat) rates from multiple identified sweat glands in 8 non-CF adults. The two types of sweating were stimulated sequentially with intradermal injections of appropriate reagents; each gland served as its own control via alternating off-on drug tests on both arms, given at weekly intervals with 3 off and 3 on tests per subject. We compared drug effects on C-sweating stimulated by either high or low concentrations of β-adrenergic cocktail, and on methacholine-stimulated M-sweating. For each subject we measured ~700 sweat volumes from ~75 glands per arm (maximum 12 readings per gland), and sweat volumes were log-transformed for statistical analysis. T-tests derived from linear mixed models (LMMs) were more conservative than the familiar paired sample t-tests, and show that ivacaftor significantly increased C-sweating stimulated by both levels of agonist, with a larger effect in the low cocktail condition; ivacaftor did not increase M-sweat. Concurrent sweat chloride tests detected no effect of ivacaftor. We conclude that ivacaftor *in vivo* increases the open channel probability (P_O_) of WT CFTR, provided it is not already maximally stimulated.

## Introduction

The genetic disease cystic fibrosis (CF) is caused by mutations that affect CFTR anion channels. Some gating mutations decrease CFTR’s average open probability (P_O_) with little or no effect on channel number. *In vitro*, the P_O_ of many gating mutations is increased by ivacaftor (VX-770)^1,2^, and when treated with orally available ivacaftor (Kalydeco) most CF patients with gating mutations show marked clinical improvement^3–6^. Ivacaftor used acutely *in vitro* also increases the P_O_ of normal human wild type (WT) CFTR^1,7,8^. However, the effect of chronic exposure to ivacaftor (24 hr or more) on WT or corrected F508del CFTR is uncertain. Additional *in vitro* studies have suggested either that it does not potentiate function of F508del or WT CFTR^9^, or that it actually decreases function^10^. At least for F508del the decrease with chronic ivacaftor is related to drug concentration, and was not observed using drug levels expected *in vivo*^11^. *In vitro* conditions differ in many respects from *in vivo* ones, and so we sought to determine if chronic oral ivacaftor dosing, taken as directed as a treatment for CFTR gating mutations, would increase, decrease or fail to affect WT CFTR function in non-CF adult subjects.

Another motivation for this work was to explore additional methods for assessing modulator effects on CFTR function *in vivo*, because no existing method is optimal across all conditions^12^. For example, FEV_1_ tests are insensitive when lung disease is either too mild or too severe. The ‘sweat test’ i.e. the chloride concentration in eccrine sweat, is a mainstay of *in vivo* CFTR functional assessment^13,14^, but also possess idiosyncratic features^15^, some of which may have contributed to unexpected results in a study of combined treatment with lumacaftor (VX-809) and ivacaftor^16^. In one arm of that study, 28 days of lumacaftor were followed by 28 days of lumacaftor + ivacaftor. While FEV_1_ improved during combination treatment vs monotherapy, sweat chloride concentration did not.

Why should this be? One possibility is that CFTR function in the sweat duct differs from its function in airway epithelia^16^. Indeed, the sweat duct has several features that suggest caution when extrapolating from sweat chloride levels to CFTR function in other organs: it is an exclusively absorptive organ; it absorbs hypertonically (salt > water); uniquely for epithelia it consists of a double layer of epithelial cells, and CFTR is expressed on both apical and basolateral membranes in the duct^17–20^. Also, sweat chloride levels have a logarithmic relation to CFTR function, making the assay progressively less sensitive at higher levels of CFTR function, being almost flat from 50–100% function^21^. Moreover, CFTR is fully activated in perfused sweat ducts, and cannot be further activated by agents that activate CFTR in other tissues^22^. Sweat duct conductance, due mainly to Cl^−^ conductance through CFTR, is among the highest known for any tissue (125 ± 14 mS/cm^2^)^23^ indicating that CFTR is abundant and probably has a high P_O_, because CFTR channels show cooperativity, with higher P_O_ values occurring when channel density is high^24^. Some combination of these features may help explain why, for ivacaftor monotherapy with G551D subjects, there was no relationship between Δ sweat chloride and Δ FEV_1_^25^.

Given these issues, we set out to assess ivacaftor effects on human WT CFTR function *in vivo* using two complementary assays: sweat chloride levels^26^ and CFTR-dependent sweat rate (C-sweat). C-sweat is rate-limited by CFTR function in the sweat gland secretory coil: it is absent in people with CF^27^ and half normal in carriers^28,29^, thus providing a near-linear readout of CFTR function. To help detect small differences in a small sample of subjects, we identified >100 individual sweat glands in each subject (>50 per arm) and used a repeated measures design where each gland served as its own control across 3 off and 3 on ivacaftor trials. In preliminary analyses of C-sweat rates, we considered only glands that were measured on all 6 tests, computed the average response on the 3 off drug tests and that on the 3 on drug tests, and then conducted a paired samples t-tests on these averages. For the main analyses, we fitted linear mixed models (LMMs) to the data from *all* glands. These LMMs included variance parameters for the random variation across glands and testing occasions (i.e., weeks), and the resulting t-tests were more conservative than the paired samples t-tests. We present both sets of results. As an additional control, for each gland we also obtained sweat rates to the muscarinic agonist methacholine (M-sweat); sweating induced by this pathway does not require CFTR^27^. To determine if the P_O_ of WT CFTR *in vivo* might be near maximal before ivacaftor (a ceiling effect) we stimulated C-sweating with two concentrations of a β-adrenergic cocktail: a saturating dose^27^, and another that was 1% of the saturating dose.

Our results show that chronic (4 day) ivacaftor treatment *in vivo* increased WT CFTR function, confirming results seen with acute ivacaftor *in vitro*^1,8^. The increase is blunted at the higher dose of agonist. Of note, we did not detect a change in sweat chloride levels for subjects on ivacaftor.

## Methods

Extended methods are presented in electronic Supplementary Material.

### Study Approval

The study (ClinicalTrials.gov: NCT02310789, 03/09/2014) was performed in accordance with all relevant guidelines/regulations, including obtaining informed consent from all participants, and was approved by Stanford University Institutional Review Board #4. After written informed consent, 8 subjects were studied: 5 non-CF adults with ‘wild type’ CFTR (no CF mutations in a screen for the 39 most common mutations) and 3 CF carriers with one CFTR mutation (all F508del).

### Study Design

**Figure 1:**
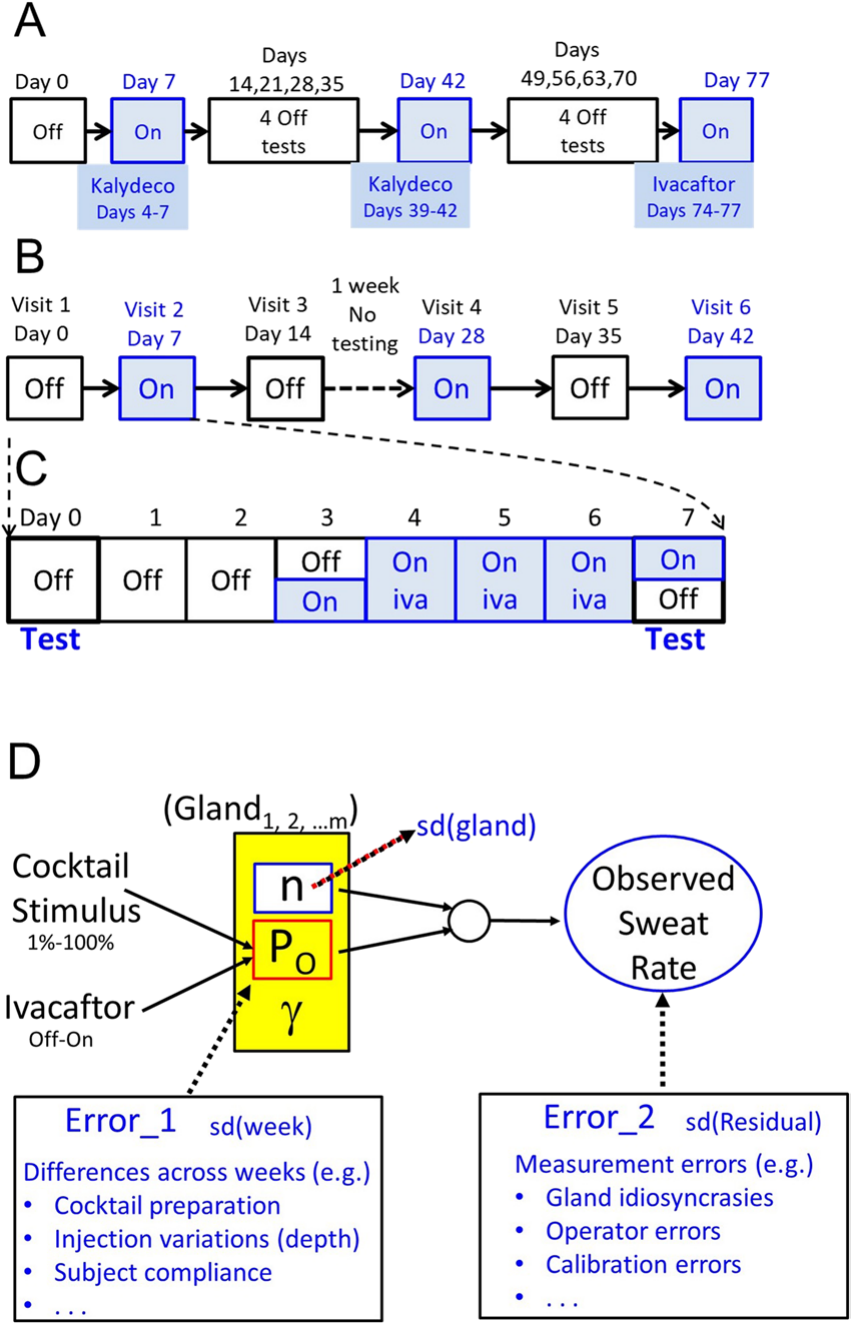
Experimental schedule and analysis scheme for linear mixed models analysis. (**A**) Schedule for Exp. 1, a pilot experiment to test for peak-trough and washout effects. (**B**) Schedule for Experiments 2 and 3. (**C**) Day-by-day schedule for on-drug test in all experiments. For Exp. 1 the first test of the study visit was done before the last dose of Kalydeco, and the 2^nd^ was done ~3 hr later. For experiments 2 and 3 the last dose of Kalydeco was taken in the morning prior to the study visit. (**D**) Each gland’s C-sweat rate is modeled as a function of n·Po·γ, with 2 fixed effects acting on Po: the cocktail stimulus concentration and presence or absence of ivacaftor. Errors of two general types (*week* and *residual*) are modeled as shown, as well as the variation, sd(gland), in *n* across glands.

We compared responses for the same identified glands off and on drug. A pilot experiment (Fig. 1a) interposed multiple weeks of off drug testing to look for washout effects, but most subjects were tested with 3 off and 3 on drug tests alternating at weekly intervals (Fig. 1b), with the last dose of ivacaftor taken on the morning of the test day (Fig. 1c). Results were assessed with linear mixed models (LMMs, Fig. 1d) as the main analysis for all data, and also with Supplementary paired t-tests.

#### Measurement of sweat secretion from identified individual glands

**Figure 2:**
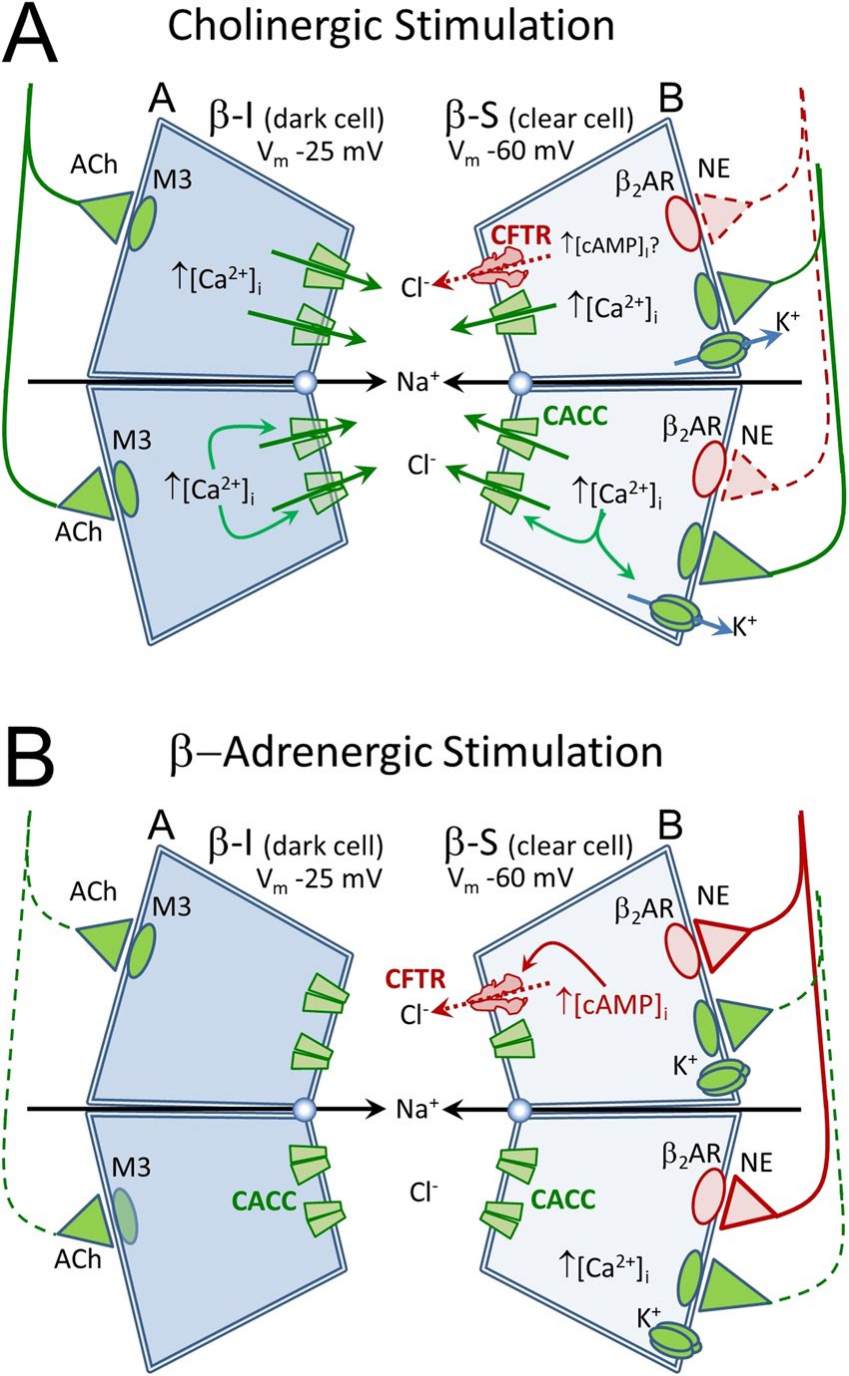
A simplified schematic diagram of ion channels responsible for anion-mediated sweat secretion. The sweat coil contains two types of anatomically distinct cell types, dark and light. Both cell types contain the machinery required to secrete anions in response to cholinergic stimulation: muscarinic receptors (M3), basolateral Ca^2+^-activated K^+^ channels and apical Ca^2+^-activated chloride channels (CACC). Clear cells also contain β2 adrenergic receptors (β_2_AR) and pathways to elevate [cAMP]_i_. (**A**) Cholinergic stimulation (mimicked by injection of methacholine) stimulates both cell types by increasing [Ca^2+^]_i_ and activating apical CaCC channels, and, in clear cells, basolateral K^+^ channels. (**B**) Adrenergic stimulation (mimicked by isoproterenol + aminophylline to elevate [cAMP]_i_ and atropine to prevent any activation of M3 receptors) activates CFTR in β-sensitive cells. Because CFTR levels are low and because no basolateral K^+^ channel is activated by isoproterenol, anion efflux and hence fluid secretion is much smaller than it is to cholinergic stimulation. (β-sensitive cells can support anion efflux because they have a higher resting gK as indicated by their more hyperpolarized membrane potential). Diagram based on experiments by M. M. Reddy, & P. M. Quinton^22,55,56^ and by Sato & Sato^57^.

**Figure 3:**
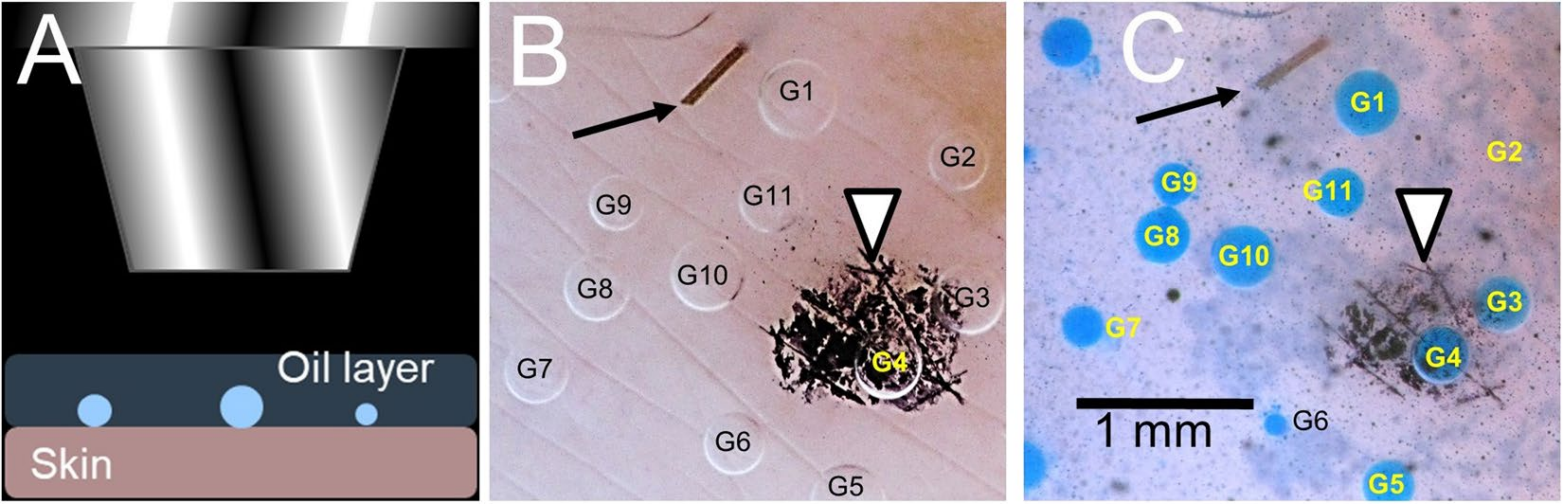
An overview of the experimental approach and examples of primary data obtained. (**A**) Imaging of sweat bubbles in oil layer. (**B**) Example of M-sweat bubbles. (**C**) Example of C-sweat bubbles stained with water soluble blue dye. Arrows in (**B**,**C**) indicate stump of cut hair. Arrowheads point to dye marker used for positioning and focusing. Glands are identified by position and numbered so they can be followed across experiments.

We used a modified version of the single gland, optical imaging assay for CFTR secretory function as described^29^. The assay depends on two parallel pathways for sweat secretion (Fig. 2): a CFTR-independent, cholinergic pathway stimulated with methacholine (‘M-sweat’) and a β-adrenergic pathway that is CFTR-dependent (‘C-sweat’). When C-sweating is expressed as a function of M-sweating, it gives a near-linear readout of CFTR function over a wide range: e.g., the C-sweat/M-sweat ratio for CF carriers is 50% that of non-CF controls, and the ratio for CF subjects is zero^28,29^. Examples of the two types of sweat are shown in Fig. 3.

### Sweat chloride collection and measurement

Sweat samples were stimulated via pilocarpine electrophoresis and collected using the Macroduct 3700 sweat collection system (Wescor Inc., Logan, UT) according to standard procedures, i.e. 5 min of stimulation and 30 min collection. One sample was collected from each subject on each study visit. Sweat chloride was measured using the QuantiChrom Chloride Assay Kit (BioAssay Systems, Hayward, CA) in accordance with the manufacturer’s instructions. Optical density (OD) of the samples and standards were read at 610 nm with a SpectraMax Plus 384 microplate reader (Molecular Devices, San Francisco, CA). Standard OD values were subtracted from the blank OD measurement, and were plotted against a curve of OD measurements of 8 Cl^−^ concentration standards from 0 to 35 (mg/dL). A slope was determined from the linear regression to convert sample OD values into final Cl^−^ concentrations (mmol/L).

### Statistics

We used two statistical approaches to evaluate the sweat secretion data. We applied linear mixed effects models (LMMs) to the log transformed data from *all* glands, using the package, lme4^30^, in the R language and environment^31^. In these multi-factorial regression analyses, results were considered significant if P ≤ 0.05. We present the lme4 syntax for the various LMMs that we used, so as to facilitate replication of our analyses by other researchers, in Electronic Supplementary Material. For a subset of glands present across all 3 off and all 3 on drug tests, we also used paired t-tests of log transformed data to test for significance within subjects, with results considered significant if P ≤ 0.001 (using Bonferroni correction for a familywise false alarm rate of ~0.05 across tests). The number of glands used in these paired-comparisons averaged 43 to 48 glands per arm (Supplementary Table 1), much less than the ~75 glands per arm that are included in the mixed models analyses. Gland responses were not measured on some trials because of merging of bubbles, poor images, or non-optimal placement of the reservoir. The requirement that a gland be measured across all M-sweat or all C-sweat tests to allow for paired t-tests resulted in ~40% of the data being ignored.

## Results

### Experiment 1 (pilot)

**S1**, male, WT/F508del, was tested at left and right arm sites, with up to 74 glands identified at each site. A linear mixed models (LMM) analysis using all glands from both arms indicated a significant increase on drug for **S1** (Table 1). **S2**, female, WT/WT, was tested using the same paradigm, but after multiple weeks of testing, she developed what appeared to be a delayed sensitivity reaction at the injection sites (itching, redness) accompanied by a precipitous drop in responding, leading us to terminate testing (Supplementary Fig. 1). LMM analysis of 203 glands from both arms showed a non-significant increase of 10′ ± 11% (Table 1). Paired t-tests for both subjects are shown in Supplementary Table 1; they indicated significant increases for one arm from each subject that did not correlate with peak-trough tests. We saw no evidence for prolonged effects in either subject. To summarize Exp. 1, the mean C-sweat rates showed small, inconsistent increases on drug for both subjects. LMM analysis of combined arm results were significant for **S1** but not **S2**, suggesting that any effect of drug on C-sweat was small enough to be partially obscured by random error in this small pilot study.

**Table 1:** Linear mixed models analyses of the change, Δ (±1 s.e.), in sweat rate due to ivacaftor (‘Off’ versus ‘On’), by subject, type of sweat and, for C-sweat, cocktail concentration (‘Full’ versus ‘1%’).

By Subj.	C sweat-Full					C sweat-1%			M-sweat		
	Δ (%)	p			*N*	Δ (%)	p	*N*	Δ (%)	p	*N*
S1 ^H^	8.14 ± 02.09***	0.0001			145	11.2 ± 4.67%	0.0653	141	5.86 ± 2.61%	0.0696	286
S2	10.14 ± 11.23	0.4581			203	not done			2.89 ± 11.24%	0.8199	203
S3 ^H^	3.86 ± 2.48	0.1607			97	10.66 ± 3.89%*	0.0369	94	0.545 ± 1.69%	0.7556	191
S4	3.36 ± 6.72	0.6405			129	not done			7.0 ± 6.73%	0.3505	129
S5	9.18 ± 10.63	0.4340			121	not done			−14.48 ± 10.6%	0.2410	121
S6 ^H^	not done					12.05 ± 4.66%	0.0565	178	−2.15 ± 4.69%	0.6690	178
S7	not done					12.4 ± 3.48%*	0.0159	181	11.73 ± 3.49%	0.0200	181
S8	not done					37.00 ± 7.35%**	0.0066	168	1.91 ± 7.44%	0.8098	168
**Pooled Data**	**C sweat-Full**					**C sweat-1%**			**M-sweat**		
	**S1–S5**					**S1, S3, S6–S8**			**S1–S8**		
	**Δ (%)**		**p**	***N***		**Δ (%)**	**p**	***N***	**Δ (%)**	**p**	***N***
Pooled	6.60 ± 2.97%*		0.0352	695		16.45 ± 2.25%***	6.86e-8	762	2.38 ± 2.66%	0.421	1208

Results are shown for each Subject, combining data from both arms (except for S3, whose data came from 1 arm), and for the pooled data. *N* refers to the number of glands used in the mixed models analyses, and significance levels are denoted by “*”, for 0.01 < *p* < 0.05; “**”, for 0.001 < *p* < 0.01; and “***”, for *p* < 0.001.

### Experiment 2, full cocktail

Because differences in peak-trough values or washout effects were not evident, in Experiment 2 we tested 3 additional subjects alternately on and off drug at weekly intervals for a total of 3 off and 3 on drug tests (Fig. 1b), with the on drug tests occurring ~4–8 hrs after their last dose of ivacaftor. One subject was tested only on one arm because the other arm lacked a visible mark needed to register gland location. Thus, experiment 2 comprised 30 tests (5 sites × 6 tests).

**S3**, Female, WT/F508del, was tested only on her left arm. LMM analysis indicated a non-significant increase on drug of 3.9 ± 2.5%, P = 0.16, 97 glands. For **S4**, Male, WT/WT, the increase on drug across both arms was 3.36 ± 6.72%, P = 0.64, 129 glands (LMM, n.s.). For **S5**, Female, WT/WT, LMM analysis of the C-sweat increase on drug across both arms was 9.2 ± 10.6%, P = 0.434, 121 glands (LMM, n.s.). Results are summarized in Table 1 for LMM analyses; paired t-tests are shown in Supplementary Table 1.

**Figure 4:**
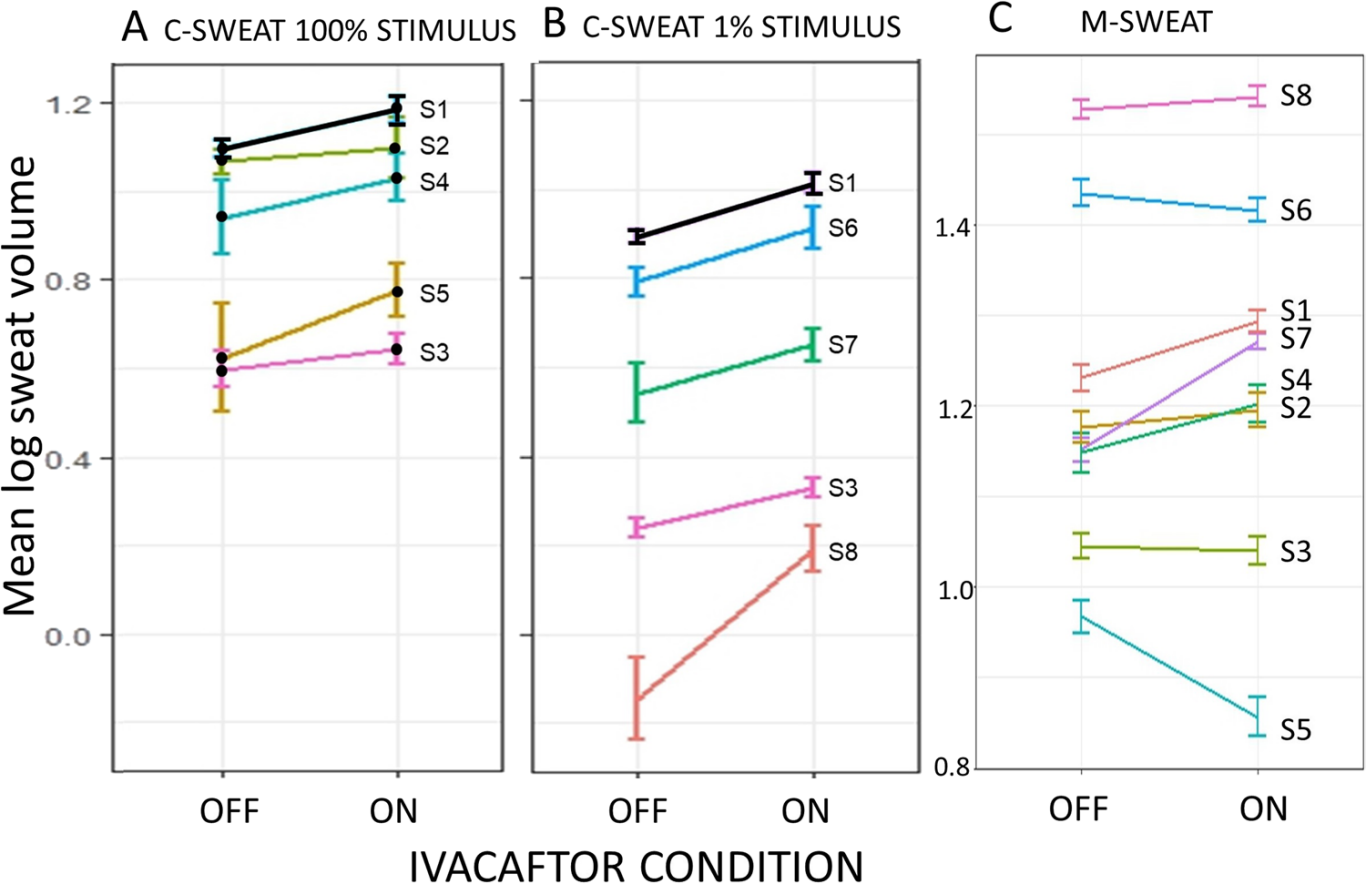
Summary of sweat rate results for all glands analyzed with linear mixed models. (**A**) Log volume of C-sweat produced by 100% cocktail off and on ivacaftor. Each point represents the (±SE) results for all glands tested in the two conditions for the subjects shown. (**B**) Log volume of C-sweat produced by 1% cocktail off and on ivacaftor. (**C**) Log volume of M-sweat produced by methacholine off and on ivacaftor. For number of glands tested and statistical methods and significance see Table 1, based on the same data but presented as Δ (%) on drug. Dashed lines and open circles indicate carriers of one F508del mutation. C-sweat volumes are based on 30 min of sweat collections and M-sweat on 10 min collection. Scales for A and B are equal.

To summarize Experiments 1 and 2, small, variable increases in C-sweat responding were produced by ivacaftor. LMM analyses (Table 1 and Fig. 4a), on pooled data (695 glands, 5 subjects) gave P = 0.035. The paired t-test analyses for each arm separately, shown in Supplementary Table 1, top panel, showed significant increases in C-sweat for 3/9 sites, non-significant increases for 5/9 sites and non-significant decrease as 1/9 sites. The small overall increase observed could result from saturation of the C-sweat rate caused by our use of a cocktail concentration that was designed to be maximal. This hypothesis was investigated in Experiment 3.

### Experiment 3: Stimulation with 1% cocktail concentration

This experiment was identical to Experiment 2, except that we reduced the concentration of isoproterenol and aminophylline in the β-adrenergic cocktail to 1% of full cocktail, while keeping the same concentration of atropine to block muscarinic receptors. Any ivacaftor potentiation of WT CFTR should be more easily discerned if glands were secreting at sub-maximal rates. Results (all data log transformed) are summarized in Table 1, Fig. 4b, and Supplementary Table 1.

**S1, M, WT/F508del** was retested with 1% cocktail. C-sweat secretion from both arms (all glands) was increased 11 ± 5% on ivacaftor (LMM, P = 0.065). Using paired t-test analyses, C-sweating increased by 18% on drug for both left (48 glands, P = 9.3E-10) and right arm sites (47 glands, P = 5.40E-07). For the combined sites, 89 of the 95 (94%) glands present across all tests showed increased C-sweat rates on drug. **S3 F, WT/F508de**l (left arm only), was also re-tested with 1% cocktail. Her increase on drug was 10.7 ± 3.9%, (LMM, 94 glands, P = 0.037); or 9% (paired t-tests, 47 glands, P = 4.55E-06), with 42/47 (89%) of the glands showing increased C-sweating. **S6 F, WT/F508del** increased 12.4 ± 3.5% on drug (LMM, 178 glands, P = 0.057). With paired t-tests, left arm C-sweat increased 16% (42 glands, P = 1.0E-06) and right arm increased 9% (66 glands, P = 6.1E-11). For both sites, 91/108 (84%) of the glands increased on drug. **S7, F, WT/WT** increased 12.4 ± 3.5% (LMM, 181 glands, P = 0.016). With paired t-tests, left arm increased 19% (57 glands, P = 3.20E-12), and right arm 4% (63 glands, P = 0.08). (Only 2 on drug tests were averaged for **S7**′s left arm site because one test produced no usable data owing to a technical failure). Across both sites 92/120 (77%) of glands increased on drug.

The last subject, **S8, M**, was recruited as a non-CF subject and like all other subjects tested negative for the 39 most common CFTR mutations. He had the lowest C-sweat/M-sweat ratio observed in this study—less than half the rate of two known carriers of CFTR mutations tested in the same way in this study. This subject’s response to ivacaftor was the largest we observed; a 37 ± 7.4% increase in C-sweat (LMM, 168 glands, P = 0.007). With paired t-tests, the left arm increased 26% (26 glands, P = 3.53E-08), and the right arm increased 44% (36 glands, P = 3.12 E-18). Across both arm sites 60 of 62 glands (97%) showed increased C-sweating on ivacaftor. The combination of low baseline C-sweating and large response to ivacaftor seen in S8 are unexplained; we cannot rule out the possibility that one allele of this subject might be a rare gating mutation that is not included in the screening panel of 39 CFTR mutations.

To summarize experiment 3, ivacaftor produced larger and more significant increases in C-sweating stimulated by 1% cocktail than it did for C-sweating stimulated with full cocktail. LMM analysis of pooled data from 762 glands showed log transformed C-sweat increasing by 16.45 ± 2.25% on drug (P = 6.86E-8, Table 1). Using paired t-tests the average increase was 18 ± 12% with all increases for single sites significant at P ≤ 0.001, except for a non-significant 4% increase on the right arm of subject **S7**.

### Ivacaftor effects on responses stimulated by full cocktail and 1% cocktail in the same glands and subjects

To assess the interaction between cocktail concentration and the level of ivacaftor more directly, we compared a set of glands at each of 3 sites from **S1** and **S3** that were measured throughout all 12 tests at both cocktail strengths (3 off/on with full and 3 off/on with 1% cocktail). For each site 28–39 glands (100 total glands) met the criterion. For all 3 sets of identical glands the effect size was larger with the reduced cocktail stimulus (Table 2), consistent with results from experiments across subjects, supporting the hypothesis that full strength cocktail increased CFTR P_O_ to near maximal values, such that further increases produced by ivacaftor would be minimized (a ceiling effect). A bonus from this comparison is that **S1** and **S3** are both CF carriers with one non-functional F508del allele and therefore ~half normal CFTR-dependent apical anion conductance. This makes it less likely that some other rate-limiting process could be responsible for the reduced effect size of ivacaftor with full-strength cocktail. This is consistent with other evidence that CFTR is rate-limiting for C-sweat^28^. Another informal test of the moderation by agonist concentration (1% vs. full) of ivacaftor’s effect on C-sweat was obtained by directly comparing the average drug effects, 16.45% and 6.60%, at the 1% and full cocktail levels, respectively. These LMM estimates have an approximate *t* distribution with about 27 and 25 degrees of freedom, respectively (using the Satterthwaite approximation). In the LMM analysis section of the online Supplementary Data, we present a conservative Z-test for the difference in drug effects. This test yields *z* = 2.539 (p = 0.011), supporting the conclusion that the drug effect is greater in the reduced cocktail stimulus.

**Table 2:** Paired t-tests on the change, Δ, in C-sweat rate due to ivacaftor (‘Off’ versus ‘On’) in identical sets of glands, for S1 (each arm) and S3 (left arm), at each cocktail concentration (‘Full’ versus ‘1%’).

Subj.	Arm	*N*	Full Cocktail stimulus			1% Cocktail stimulus		
			*n*↑	Δ (%)	P value	*n*↑	Δ (%)	P value
S1	L	28	17/28	2%	0.44	25/28	18%	1E-05
S1	R	33	30/33	8%	0.008	30/33	14%	2E-04
S3	L	39	27/39	3%	0.223	30/39	10%	3E-05

*N* refers to the number of glands used in the t-tests, and *n*↑ refers to the fraction of glands showing an increase due to ivacaftor. The estimates of Δ are in line with those in Table 1, but the p-values are likely too small, as argued in the Discussion.

### Methacholine-stimulated sweat rates off and on ivacaftor

Every C-sweat test was preceded by an M-sweat test at the same site (see Methods). To assess ivacaftor effects on M-sweating we combined results across all experiments because stimulation of M-sweat followed the same protocol throughout, giving 18 comparisons from 8 subjects, based on 52 separate tests (each comparison based on triplicate tests except for **S2**, duplicate only) with 1457 identified glands. No significant increase of M-sweat secretion was detected in the presence of ivacaftor: the increase across all subjects was 2.1 ± 2.2% (P = 0.34, n.s., LMM analysis, Table 1, Column 3), and paired t-tests gave erratic results (−31% to + 31%, Supplementary Table 2) with no significant overall trend. The inability to see an ivacaftor effect on M-sweating in non-CF subjects is consistent with the very small contribution of CFTR to apical anion conductance during cholinergic stimulation (Fig. 2A).

### Sweat chloride values off and on ivacaftor

**Figure 5:**
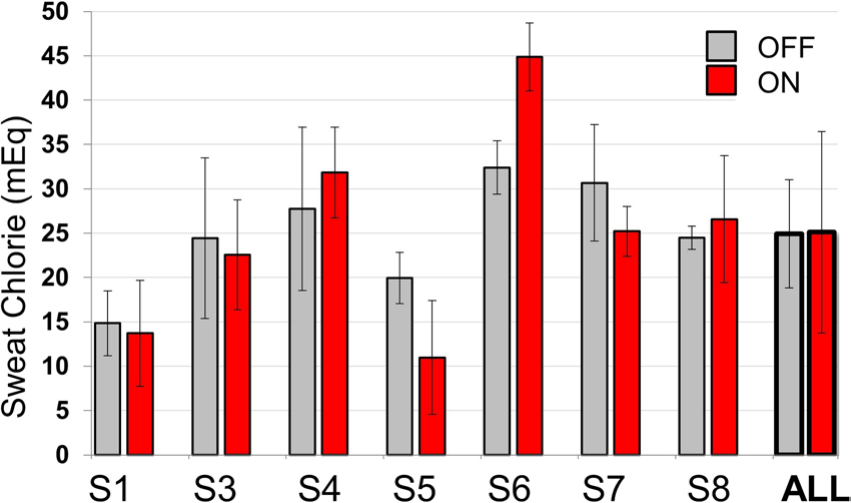
Average sweat chloride values off and on Kalydeco for each subject and averaged for all subjects. For S1, S2 and S4-S8, each gray bar represents the mean ± SD of 3 off drug tests and each red bar the mean ± SD of 3 on-drug tests. S3 was tested in both experiments 2 and 3 so each bar represents the mean of 6 tests. Sweat chloride values were not obtained for S2 who was only run in the pilot experiment (Exp. 1). Grand means for all 7 subjects are shown with bold bars labeled ‘All’.

On each study visit in experiments 2 and 3, Macroduct sweat chloride testing was conducted on one arm, providing 3 off drug and 3 on drug measurements for each subject, except for **S3**, who was run in both experiments and so had 6 tests in each condition. Results are shown in Fig. 5. Four subjects showed decreases on drug and 3 showed increases, with no net change overall. The average values off and on drug were 24.9 ± 6.1 and 25.1 ± 11.4 respectively, yielding a difference that was not significant (P > 0.92) for either the paired t-test based on the 7 difference scores (1 for each subject), or the LMM based on the 48 measurements from the 7 subjects.

## Discussion

This research set out to answer five related questions. Two main questions were whether four days of oral ivacaftor (Kalydeco) would produce an increase in WT CFTR function^1,8^, a decrease^10^, or have no effect^9^, and whether its effect would be altered by the concentration of the β-adrenergic cocktail used to stimulate CFTR. We also asked if ivacaftor would increase M-sweat rates, if the sweat rate assay was more sensitive for detecting changes in WT function than the sweat chloride assay, and if our conclusions held across the two statistical approaches.

As assessed with C-sweat rates, ivacaftor did increase WT CFTR function *in vivo*, and the effect size was increased using a weaker β-adrenergic cocktail to stimulate CFTR. We did not detect a significant effect of ivacaftor on M-sweat or on sweat chloride levels. The pattern of drug effects for *sweat rates* held both for linear mixed models (LMM) analyses and paired t-tests. The LMM analyses allowed for the estimation of week-to-week variability, separately from that of measurement error, and this led to a more conservative test of the drug effect. In other words, the paired t-ratio was inflated because it was based on a standard error of estimate that ignored week-to-week variability and was, therefore, too small. Further, the paired t-tests required about 40% of the glands to be ignored (because they weren’t measured in all tests), and this decreased the power of the test. For these reasons, our conclusions about sweat rates rely mainly on the LMM results. When assessing the drug effect on *sweat chloride* measurements, however, week-to-week variability was confounded with measurement error, because there was only 1 measurement per week in each drug condition, and the paired t-test was likely as conservative as the LMM. Not surprisingly, the two approaches yielded nearly identical results.

### Ivacaftor potentiated WT CFTR *in vivo* with evidence for a ceiling effect

Ivacaftor potentiation was most evident when sub-maximal concentrations of the β-adrenergic cocktail were used (Table 1 and Fig. 4), presumably because the saturating concentration of β-adrenergic cocktail^27^, pushed CFTR P_O_ near to its (*in vivo*) maximum. The mean increase produced by ivacaftor (prior to log transformation) was 13 ± 16% for glands stimulated with full cocktail, vs. 41 ± 31% for glands stimulated with a sub-maximal β-adrenergic cocktail. The 13% increase is smaller than observed *in vitro* with acute addition of ivacaftor to forskolin-stimulated cells, where increases of 50–100% have been observed^2,7,32^. In addition to acute vs. chronic addition, many other differences between the *in vivo* and *in vitro* experiments might explain the quantitative differences. The present approach is important because it shows that ivacaftor potentiates WT CFTR *in vivo* when used as directed. Because of evidence that smoking suppresses CFTR^33^, this finding supports the proposal that ivacaftor could be useful for the treatment of smoking related COPD^34–39^.

### An ivacaftor effect was not detected on sweat chloride levels

Sweat chloride levels were not decreased by ivacaftor in these non-CF subjects. This is not surprising for two reasons. First, sweat chloride provides a logarithmic readout of CFTR function^21^, falling steeply from ~100 mM when CFTR function is zero to ~40 mM when CFTR function is ~10% and becoming less sensitive thereafter, so that non-CF subjects can barely be distinguished from CF carriers, who have only 50% WT CFTR function^40^. The increase in WT CFTR function we saw is predicted to decrease sweat chloride values by only ~3 mM (extrapolated from Fig. 14 in Char *et al*.^21^), consistent with the nearly identical values that we observed off and on drug. Second, it is possible that ivacaftor cannot increase WT CFTR function in the duct, where CFTR is fully activated^22^, and its high density may push its P_O_ even higher^24^.

### Ivacaftor did not significantly alter M-sweat

A recurring question is whether cholinergically-stimulated M-sweat, which persists in CF subjects, is completely CFTR-independent. Although we did not detect a significant ivacaftor effect on M-sweating (Table 1 and Fig. 4c), it is likely that CFTR does make a minor contribution to M-sweating. CFTR and calcium-activated chloride channels are co-localized in the same sweat coil secretory cells^41^ (and shown in Fig. 2), and reduced cholinergic sweat rates are seen for CF subjects in some^27–29^, but not all^42^ studies. CFTR contributes to cholinergically-mediated fluid secretion in several tissues and species^43–49^, and stimulating M3 muscarinic receptors in CFTR-transfected BHK cells^50^ or P2Y receptors via apical UTP in well-differentiated airway cells^51^ activates CFTR. Ivacaftor provides a tool to measure the participation of CFTR in cholinergically induced M-sweat in CF subjects whose CFTR mutations show large increases to ivacaftor. For example, in an R117H-7T subject where ivacaftor produced an estimated 3–7 fold increase in P_O_, M-sweating was also significantly increased^52^. In these non-CF subjects we may have failed to detect a contribution of CFTR on M-sweating because ivacaftor increased CFTR function by 13–41% in these experiments, versus the ~500% increase seen in the R117H-7T subject^52^.

### Advantages/disadvantages of the assay and limitations of the study design

This assay is technically challenging, but provides an accurate and near-linear assessment of CFTR function *in vivo*, and multiple measures obtained through repeat visits provide sufficient data to detect small differences in function using a small set of subjects. The technical demands will likely limit its use to research settings, where the approach can be useful in calibrating biomarkers used in clinical settings (see for example Graeber *et al*.^53^).

This research benefitted from several distinct advantages that helped us estimate the relevant fixed effects (e.g., the drug effect on C-sweat) with greater accuracy by taking account of random variation across glands, testing occasions (weeks), and subjects. First, ivacaftor is a highly specific drug that binds CFTR directly^7^. Second, C-sweat depends absolutely on CFTR^27^ and varies nearly linearly with CFTR function^28,29^. Third, we used a method in which two sets of identified glands (left and right arms) were independently stimulated on multiple test days for all but one subject, and for comparison we also activated a separate cholinergic pathway that is not CFTR-dependent.

This study also had important limitations. It was not a placebo controlled, double blind trial. Also, it is clear in retrospect that the experiments looking for peak-trough and washout effects would have been more sensitive if they had been run using 1% cocktail. The small number of subjects could also be considered a limitation, but one purpose of the study was to determine if the multiple gland, within-subject design would reveal a clear signal with a small number of subjects. In this respect, our study was reasonably successful.

### Summary and Conclusions

In summary, 4 days of oral ivacaftor produced increases in WT CFTR function that were most reliably detected with C-sweat rate assays conducted with sub-maximal β-adrenergic stimuli, less reliably detected with C-sweat rate assays conducted with maximal stimuli, and not detected with M-sweat measurements or sweat chloride assays. When a drug that is efficacious *in vitro* fails to show *in vivo* effects it is commonly interpreted to mean that the drug has failed, but the present results show that real effects can be masked by features of the *in vivo* measurements used. This point applies especially to the ‘gold standard’ measure of FEV1, which is strongly influenced by environmental factors and is insensitive to improved CFTR function especially at early or late stages of disease. For a discussion of biomarkers as surrogate endpoints, see De Boeck *et al*.^54^; and, for a comparison of various biomarker responses to CFTR modulators, see Graeber *et al*.^53^.

## Electronic supplementary material

Dataset 1
