# Supplementary material for: Sweat rate analysis of ivacaftor potentiation of CFTR in non-CF adults
Source: Sci Rep. 2018 Nov 2;8:16233. doi: 10.1038/s41598-018-34308-8 (PMC6214959; doi:10.1038/s41598-018-34308-8)
Supplement: Supplementary file 1 — Dataset 1 [file 41598_2018_34308_MOESM1_ESM.pdf]

Electronic Supplementary Material

Sweat rate analysis of ivacaftor potentiation of CFTR in non-CF adults

Jeeyeon Kim[1], Miesha Farahmand[1], Colleen Dunn[2], Carlos E. Milla[2], Rina Imari Horii[3], Ewart A. C. Thomas[3] Richard B. Moss[2], & Jeffrey J. Wine[1,2,3,*]

[1]Cystic Fibrosis Research Laboratory, Stanford University, Stanford, CA, 94305, USA

[2]Department of Pediatrics, Stanford University School of Medicine, Stanford, CA, 94305, USA

[3]Department of Psychology, Stanford University, Stanford, CA, 94305, USA

CONTENTS

Details of methods

Linear Mixed Models analysis of data using lme4

Supplementary information: Anomalous data points

Supplementary information: Data validation

Supplementary Figure 1: test by test results for experiment 1.

Supplementary Figure 2. Example of Data validation

Supplementary Table 1: Paired t-tests for C-sweat responses OFF and ON ivacaftor.

Supplementary Table 2: M-sweat rates off and on ivacaftor.

Supplementary Table 3. Estimates of random effects derived from Linear Mixed Models analyses

References

Details of Methods

Subjects. The study was performed in accordance with all relevant guidelines/regulations, including obtaining informed consent from all participants, and was approved by Stanford University Institutional Review Board # 4 (Jennifer A. Howden, Manager). The Stanford University IRBs are in compliance with Good Clinical Practices (ICH/GCP) as consistent with US Food and Drug Administration Code of Federal Regulations (21 CFR 50 and 56) and DHHS Regulations (45 CFR Part 46). Informed consent was obtained from the adult participants (no children were enrolled) prior to any research related intervention. The informed consent process was documented within each participant's source documentation. The study was registered with ClinicalTrials.gov: NCT02310789, 03/09/2014. After written informed consent, 8 subjects were studied: 5 non-CF adults with 'wild type' CFTR (no CF mutations in a screen for the 39 most common mutations) and 3 adult CF carriers with one CFTR mutation (all F508del).

Stimulation and imaging of sweat secretion. We used a modified version of the single gland, optical imaging assay for CFTR secretory function as described[1]. In brief, a specific, identified region of skin on the volar forearm was injected intradermally with methacholine to stimulate CFTR-independent sweating (M-sweat) which was measured for 10 min; the area was cleaned and reinjected with a cocktail of isoproterenol, aminophylline, and atropine in lactated Ringer's to block M-sweating and produce C-sweating. For both types of sweating bubbles of sweat from single glands were captured in an oil layer, visualized by oblique lighting or dye-partitioning[1], and digitally imaged at 30 sec intervals. Individual glands were identified by location relative to landmarks and one another (gland constellations). For each identified gland the increases in M- and C-sweat volumes over time were recorded and average M- and C-sweat rates/min were calculated by dividing the final sweat volumes for each gland by 10 or 30 min respectively. In the plots of M- and C-sweat correlations, each point represents the average rate for a single identified gland based on all trials where the gland's secretion was measured.

Conditions for M-sweating were modified in 2 ways to reduce the merging of M-sweat bubbles. We used 0.05 ml of a 1 μM solution of methacholine, which is 1/2 the volume used previously, and M-sweating was monitored for only 10 min, instead of the 15 min used in prior tests[1,2]. C-sweating was monitored for 30 min in response to 0.1 ml of cocktail as before. Full-strength cocktail was used for experiments 1 and 2. In Exp. 3 the concentrations of isoproterenol and aminophylline were reduced to 1% of their normal concentrations, with the atropine level left at full strength. In prior work with 1% cocktail the C-sweat rates were reduced to 26% of control values[1]. Substantial levels of C-sweating were observed in these non-CF subjects even with the reduced β-adrenergic cocktail, so that correction for sweat losses[1,2] was not required.

Reagents Kalydeco (150 mg ivacaftor tablets, supplied by Vertex Pharmaceuticals), Methacholine Chloride (Methapharm, Ontario, Canada), Isoproterenol HCl, Aminophylline, lactated Ringer's (Hospira, Lake Forest, IL), and Atropine Sulfate (American Reagent) were obtained from Stanford University Hospital Pharmacy. Heavy mineral oil was obtained from

EMD Chemicals, Gibbstown, NJ, and was water-saturated before use as previously described[1]. Erioglaucine disodium salt (CAS No. 3844-45-9) was obtained from Sigma.

Linear Mixed Models analysis of data using lme4

For a comprehensive analysis using multi-factor designs, we used linear mixed effects models that were analyzed using the package `lme4`[3], in the R language and environment[4]. These regression analyses were conducted on log transformed data from *all* glands, with results considered significant if $P \leq 0.05$. We present here the `lme4` syntax for the various LMMs that we used, so as to facilitate replication of our analyses by other researchers.

In brief, we focused on 3 fixed effects, namely, those of Drug (Kalydeco: On vs. Off), Sweat (C vs. M), and Conc (1% vs. 100%), where Conc refers to the concentration of the β-adrenergic cocktail used to stimulate C-sweat. In some analyses, we included also the effect of Arm as a covariate. We asked: Does Kalydeco significantly increase or decrease the level of C-sweat? Does Kalydeco significantly increase or decrease the level of M-sweat? Is the effect of Kalydeco greater on C-sweat than on M-sweat? We examined these questions by analyzing the data separately for Expts 1-2 (i.e., Conc = 100%), and for Expt 3 (Conc = 1%); and separately for each Subject. To estimate the overall effect of Kalydeco, we pooled the data across Subjects and across Arms, and analyzed the pooled data separately for each level of Conc.

Preliminary analyses suggested that for some, but not all, subjects: (i) there was a significant Drug * Sweat interaction, and (ii) there was appreciable variation across glands in average C-sweat response off-drug, as well as in the difference between average C-sweat and M-sweat. Accordingly, all models tested included the Drug * Sweat interaction as a fixed effect, and the Gland * Sweat interaction as a random effect. (The function, `anova()`, within the `lme4` package allows for the testing of nested models using likelihood ratio tests. In this way, we can decide, e.g., if a model with an interaction term fits the data significantly better than the same model without the interaction term.) In addition, all models tested included a random effect for Week. Thus, the basic model used can be expressed in `lme4` syntax as:

```
LogVolume ~ Sweat * Drug + (1 | Week) + (1 + Sweat | Gland).   (1)
```

For the 7 Ss who provided data from both arms at either concentration, the basic model was expanded to include Arm as a covariate, with Gland nested within Arm:

```
LogVolume ~ Arm + Sweat * Drug + (1 | Week) + (1 + Sweat | Arm / Gland).   (2)
```

The remaining subject, **S3**, provided data at both levels of Conc from the left arm only, and the basic model in Eq. (1) was used on these data.

To estimate the overall effect of Kalydeco, we pooled the data across Subjects and across Arms, and analyzed the pooled data separately for each level of Conc. In effect, we treated Arm (relabeled as SubjectArm), rather than Subject, as the randomly sampled unit of analysis, and

treated Gland as nested within SubjectArm. The version of the basic model used on the pooled data at each level of Conc was:

```
LogVolume ~ Sweat * Drug + (1 | Week) + (1 + Sweat | SubjectArm / Gland). (3)
```

The foregoing analyses allow for formal tests of the fixed effects of Drug and Sweat on C-sweat and on M-sweat, at each level of Conc, and for an informal comparison of the drug effect between the 2 levels of Conc. Our informal estimation of the moderation by Conc of the drug effect on C-sweat proceeded as follows. The LMMs yielded estimates for the full and 1% cocktail levels, respectively, of (i) the drug effects, D_1 and D_2, (ii) standard errors of estimate, s_1 and s_2, and (iii) the Satterthwaite approximation to the degrees of freedom, n_1 and n_2. Under the null hypothesis, the ratio, D_i/s_i, $i = 1, 2$, has the t distribution with n_i degrees of freedom, and, therefore, has a variance of $n_i/(n_i - 2)$. As is well-known, for "large" n_i, this t distribution of D_i/s_i is well approximated by the Normal distribution with mean, 0, and variance, $n_i/(n_i - 2)$. It follows that, to a good approximation, D_i is Normally distributed with mean, 0, and variance, $[(s_i^2 * n_i)/(n_i - 2)]$; and the difference, $D_2 - D_1$, is also Normally distributed with a mean of 0. Computing the variance of $D_2 - D_1$ is complicated by the fact that 2 Subjects, **S1** and **S3**, contribute observations to both D_1 and D_2, and so introduce a correlation between the 2 estimates. Since this correlation is most likely to be positive, we can proceed conservatively by noting that the variance of $D_2 - D_1$ is, therefore, most likely to be less than the sum of the variances, var(D_1) + var(D_2), and then using this sum as our estimate of the variance of $D_2 - D_1$. The LMM estimates for (D_i, s_i, n_i) were (6.60%, 2.97%, 25) and (16.45%, 2.25%, 27), for $i = 1$ and 2, respectively. The upper bound for the variance of $D_2 - D_1$ is calculated to be $15.055 = 3.88^2$; and this yields a z-score of $z = (D_2 - D_1)/3.88 = 9.85/3.88 = 2.539$ (p = 0.011). This result of our conservative approach supports the conclusion that the drug effect is greater in the reduced cocktail stimulus.

Although these analyses based on Eqs. (1)-(3) yield answers to all of the questions we posed in our study, we ran additional tests for two reasons. First, because the administration of the β-adrenergic cocktail followed M-sweat measurements on testing days, Conc cannot affect levels of M-sweat. Accordingly, we pooled M-sweat measurements across levels of Conc and levels of SubjectArm in order to better describe the drug effects on M-sweat. For this analysis, we used a reduced version of the basic model:

```
LogVolume ~ Drug + (1 | Week) + (1 | SubjectArm / Gland).        (4)
```

We found that the drug effect on M-sweat was not statistically significant (b = 0.0238, p = 0.421; see the bottom row of Table 1). This result is consistent with those based on the models in Eqs. (1)-(3).

Second, to generate formal tests of whether the level of Conc affects the size of the drug effect, we considered only C-sweat responses, pooled across both levels of Conc and both levels of Drug, and tested the Conc * Drug interaction for statistical significance. We performed:

(i) Three within-Subject analyses using the data from each arm of **S1** and from **S3**, and the model,

```
LogVolume ~ Conc * Drug + (1 | Week) + (1 | Gland).        (5)
```

(ii) A pooled analysis using the data from **S1** and **S3**, and expanding the model in Eq. (5) appropriately:

```
LogVolume ~ Conc * Drug + (1 | Week) + (1 | SubjectArm / Gland).    (6)
```

And

(iii) A pooled between-Subject analysis using the data from **S2, S4, S5, S6, S7** and **S8**, and the model in Eq. (6).

Of the three analyses in (i), one yielded a significant Conc * Drug interaction in the predicted direction (b = -0.0013, p = 0.005), one yielded a marginally significant interaction (b = -0.0006, p = 0.071), and one yielded an insignificant interaction (b = 0.0004, p = 0.375). The pooled analysis in (ii) yielded a marginally significant interaction (b = -0.046, p = 0.058); and the pooled between-Subject analysis in (iii) yielded an insignificant interaction in the predicted direction (b = -0.123, p = 0.144). On balance, these results are consistent with our earlier conclusion that the effect of ivacaftor is somewhat weaker in the full concentration condition, although the moderation by Conc is itself weak.

Finally, we obtained 3 off- and 3 on-drug measurements of sweat chloride from each Subject, except for **S3**, from whom we obtained 6 off- and 6 on-drug measurements. For the paired samples t-test, we computed the average off- and average on-drug response for each of the 7 Subjects, and based the test on these 7 pairs. For the corresponding LMM analysis, we analyzed all 48 measurements using the following model:

```
Chloride ~ Drug + (1 | Subject).       (7)
```

Both approaches led to the conclusion that ivacaftor does not affect sweat chloride levels.

Estimates of the random effects. The LMMs used herein yield estimates of the variability due to Gland, Week and Subject, as well as that due to measurement error. For some Subjects, the variation across glands was best accounted for by 3 parameters: the standard deviation of the average C-sweat response off-drug (the “intercept”), the standard deviation of the difference between average C-sweat and average M-sweat responses off-drug (the “slope”), and the correlation between intercept and slope. To obtain reliable estimates of these 3 parameters, we recoded Sweat as -1 for C-sweat and +1 for M-sweat. The estimates derived from Models 1-3 of these random effects, i.e., the standard deviations (s.d.’s) and correlations, are given in Supplementary Table 3. It can be seen there that the error s.d. is about 0.25, with a range of (0.19, 0.32) across Subjects. With respect to variability across glands, the s.d. of the average response per gland (the intercept) is about 0.27, with a range of (0.19, 0.38), whereas that of the difference between M-sweat and C-sweat levels (the slope) is about 0.11, with a range of (0.06, 0.16). The correlation across glands between intercept and slope is about -0.50, with a range of (-1.0, 0.38). Analyses of the pooled data across Subjects also yielded estimates of the standard deviation across Subjects of the Subject random effect. These estimates are 0.238 and 0.180 when Conc = 1% and

Full, respectively, and are not included in Supplementary Table 3. Finally, the standard deviation of the Week random effect is about 0.07, with a range of (0.0, 0.13).

To summarize, the variability in average response across glands within a Subject, the variability in average response across Subjects, and the variability across tests due to measurement error are all approximately equal, in the range of 0.20 to 0.25. Variability across glands in their sensitivity to type of sweat is about half as much, and the variability across Weeks is about half as much again. It is heartening that the variability due to Weeks is low relative to measurement error, because that suggests that we achieved good control over the testing procedure from week to week. The fact that glands differ from each other as much as Subjects differ from each other suggests that it may be worthwhile to explore the biophysical basis for this considerable variation across glands – in the same way that the vexing variability across patients has led researchers to explore the factors that lead to individual differences. More generally, the magnitudes of these random effects can be used to explore theoretically various properties of these linear mixed models, such as, the *precision of estimate* for the effect of ivacaftor, and the dependence of this precision on design features, such as the number of glands and the number of measurements per gland. We plan to present such explorations in a separate article.

Supplementary information: Anomalous data points

Three subjects, **S2, S5** and **S7**, gave results that were anomalous in the overall context when assessed with paired t-tests (Supplementary Table 2). **S2** showed a large drop in responding after 10 weeks of testing in the extended pilot experiment, accompanied by evidence for delayed hypersensitivity. **S5** was the only subject to show a decrease in C-sweat on-drug (only one arm and not a significant decrease) as well as significant decreases in M sweat on both arms. The decreased C-sweat seen on-drug for the right arm site was the result of a marked decrease in responding for the 6th (last) test at the site, which was 24% of the average of the previous 5 trials. The M-sweat responses on the last trial (both arms) were also smaller on than on any previous trial, causing the only significant decreases in M-sweat on-drug seen across the 18 sessions. If only the first 4 trials were averaged for this subject (2 off and 2 on), no significant difference on-drug is seen. As suggested for **S2**, a delayed hypersensitivity response might have developed over the testing period, but we did not pursue this explanation for either subject, because biopsies to confirm or rule out hypersensitivity were considered to be ethically unwarranted. **S7** showed a large apparent increase in M-sweating for the right arm only, which was traced to one aberrantly low test off drug. Removing this test from the analysis converted the significant increase to a non-significant decrease. The pattern of these anomalies make it clear that they arise from subject/testing variability, rather than variability in drug effects.

Supplementary information: Data validation

Data validation is a term usually applied to procedures designed to catch errors in programs and spreadsheets. We use it here to evaluate the magnitude of random factors and to expose errors in our testing. The experimental design has several features that facilitate data validation. First, we used a repeated measures, within-subject design. In particular, for each site on an arm we

measured C-sweat and M-sweat responses from multiple individually identified glands across 6 tests, at weekly intervals, under alternating off and on drug conditions. Second, for all but one subject, we tested both arms independently. Third, we tested multiple subjects. Fourth, we stimulated C-sweat with two levels of agonists, one designed to be maximal and the other 1% of that concentration. All of these factors were included in our analyses through the use of linear mixed effects models.

In addition, we ran cross-correlations for all the glands used in the paired comparisons that generated Supplementary Table 1. Each subject was tested 6 times per site: (3 off and 3 on ivacaftor), so for 6 pairwise comparisons we have $[(6-1)\cdot 6]/2 = 15$ comparisons. We then averaged the r values for all 15 cross correlations as a measure of test consistency: mean r values ± SDs are shown in the column labeled 'test consistency' in this Table. For full cocktail, the average r across all subjects was 0.67 ± 0.04 and for 1% cocktail it was 0.79 ± 0.13. It can be seen that when one arm showed less significant increases on ivacaftor it also typically had a lower test consistency, indicating that something about the test, and not the drug effect, was likely responsible for the difference. The process is illustrated in Supplemental Figure 2 for an example in which it identified a single outlier test (2/19 test on right arm). We did not perform this analysis for M-sweat.

Supplementary Figure 1: test by test results for experiment 1.

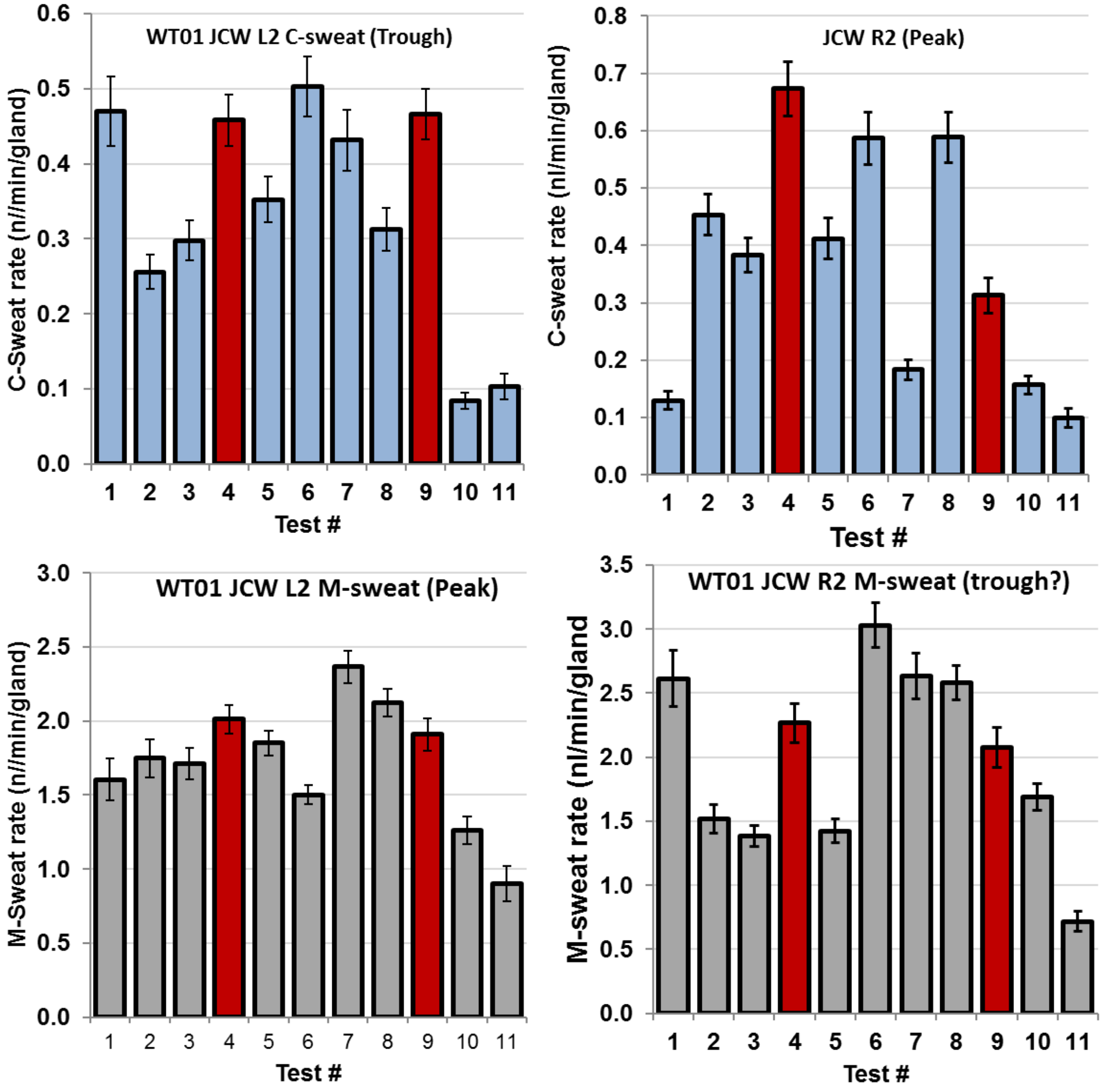

Supplementary Figure 1. Subject 2, WT/WT. Average C-sweat rates (A, B) and M-sweat rates (C, D) for the number of glands shown on weeks 1-11. Red bars show results on drug. (A) C-sweat, site L2, taken ~12 hrs after last dose (predicted trough). (B) C-sweat, Site R2, studied ~4 hrs after last dose (predicted peak). (C) M-sweat, site L2, taken ~12 hrs after last dose (predicted trough). (D) M-sweat, Site R2, studied ~4 hrs after last dose (predicted peak). Testing stopped after week 11 because of what appeared to be a delayed hypersensitivity reaction at the test site.

Supplementary Figure 2. Example of Data validation

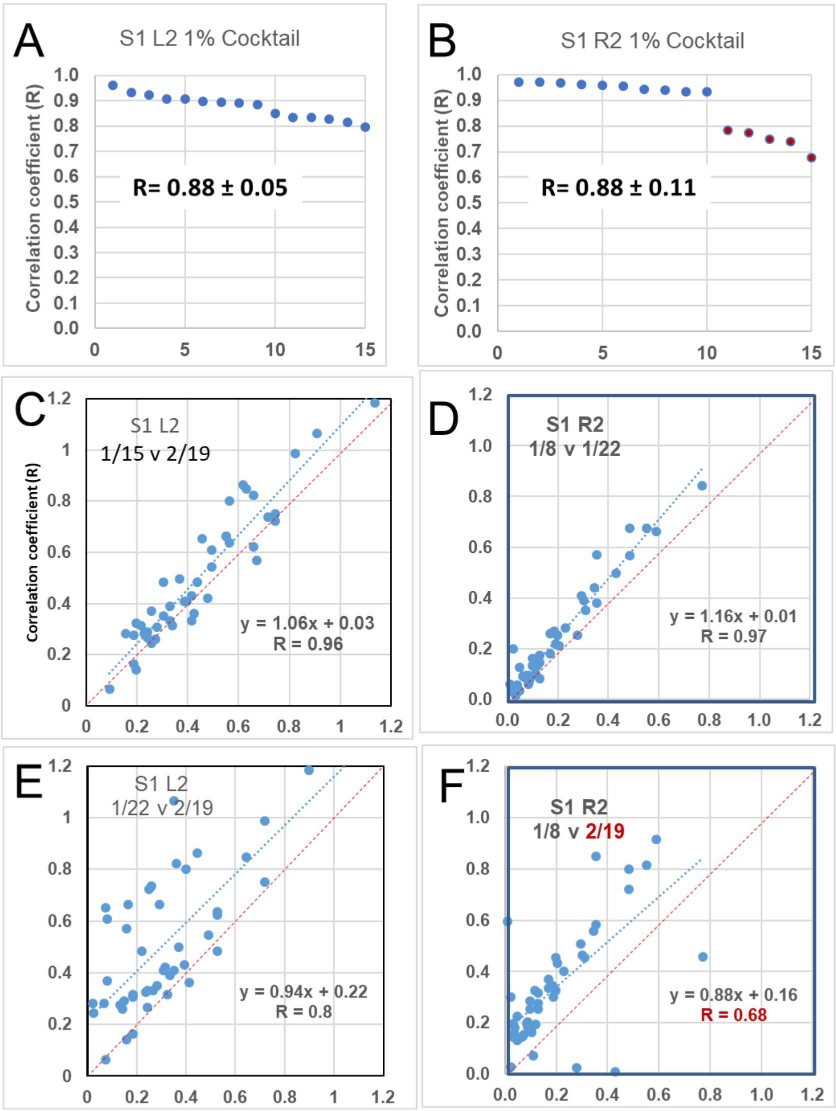

Supplementary Figure 2. Cross-correlations for 1% C-sweat responses of identified sweat glands across 6 tests for S1. A, B plot R values for each of 15 cross-correlations for left (A) and right sites, rank ordered from highest (R=0.97) to lowest (R=0.68), with average R shown in bold. Graphs C, D show best correlations for left and right sites respectively; E and F show the worst correlations. Experiment on 2/19 for site R2 had several poorly responding glands, (F) leading to the marked drop in cross correlations with the other tests (red points in B)

Supplementary Table 1: Paired t-tests for C-sweat responses OFF and ON ivacaftor.

	Exp #	Subj	GT	Sex	Arm	n total SGs	SGs all tests	Mean Rate OFF (± SD)	Mean Rate ON (± SD)	Δ rate (%)	Δ log vol (%)	Sig.	test consistency
	**C-sweat full cocktail**							±	±			P=	R ± SD
1	**Ex1**	**S1**	Hz	M	L Tr	72	36	0.68 ± 0.05	0.73 ± 0.13	8%	3%	0.1993	0.69 ± 0.15
2	**Ex1**	**S1**	Hz	M	R Pk	61	42	0.48 ± 0.02	0.56 ± 0.08	16%	8%	4.89E-04	0.76 ± 0.09
3	**Ex1**	**S2**	WT	F	L Tr	112	82	0.36 ± 0.04	0.50 ± 0.05	38%	18%	2.47E-06	0.48 ± 0.13
4	**Ex1**	**S2**	WT	F	R Pk	90	61	0.54 ± 0.18	0.55 ± 0.21	2%	1%	0.7863	0.45 ± 0.14
5	Ex2	S3	Hz	F	L	85	53	0.20 ± 0.03	0.22 ± 0.04	8%	4%	0.063	0.79 ± 0.14
6	Ex2	S4	WT	F	L	73	48	0.48 ± 0.07	0.56 ± 0.12	17%	7%	4.90E-04	0.72 ± 0.06
7	Ex2	S4	WT	F	R	49	28	0.44 ± 0.05	0.49 ± 0.13	11%	3%	0.312	0.69 ± 0.18
8	Ex2	S5	WT	M	L	56	21	0.3 ± 0.05	0.40 ± 0.07	33%	12%	0.0016	0.75 ± 0.09
9	Ex2	S5	WT	M	R	41	14	0.23 ± 0.19	0.20 ± 0.10	-15%	-6%	0.259	0.39 ± 0.20
	Means:					71.0	43	0.41 ± 0.15	0.47 ± 0.17	13±16	6±7		0.67 ± 0.04
	Totals					639	385						
	**C-sweat 1% Cocktail**											P=	R ± SD
1	**Ex3**	**S1**	Hz	M	L	72	48	0.33 ± 0.00	0.51 ± 0.06	52%	18%	9.34E-10	0.88 ± 0.05
2	**Ex3**	**S1**	Hz	M	R	61	47	0.22 ± 0.03	0.29 ± 0.08	35%	18%	5.40E-07	0.88 ± 0.11
3	**Ex3**	**S3**	Hz	F	L	72	47	0.10 ± 0.01	0.12 ± 0.01	18%	9%	4.55E-06	0.85 ± 0.09
4	**Ex3**	**S6**	Hz	F	L	73	42	0.31 ± 0.08	0.43 ± 0.02	39%	16%	1.02E-06	0.84 ± 0.06
5	**Ex3**	**S6**	Hz	F	R	70	66	0.37 ± 0.09	0.42 ± 0.06	13%	9%	6.06E-11	0.90 ± 0.03
6	**Ex3**	**S7**	WT	F	L	93	57	0.15 ± 0.04	0.22 ± 0.01	51%	19%	3.20E-12	0.73 ± 0.10
7	**Ex3**	**S7**	WT	F	R	87	63	0.17 ± 0.05	0.18 ± 0.02	4%	4%	0.08	0.62 ± 0.17
8	**Ex3**	**S8**	WT	M	L	58	25	0.05 ± 0.03	0.07 ± 0.01	50%	26%	3.53E-08	0.53 ± 0.19
9	**Ex3**	**S8**	WT	M	R	76	36	0.06 ± 0.04	0.13 ± 0.03	####	44%	3.18E-18	0.84 ± 0.06
	Means:					73.6	48	0.20 ± 0.12	0.26 ± 0.16	41±31	18±12		0.79 ± 0.13
	totals					662	431						

Each line shows experimental results for L or R arm site. Top section of table is responses to full cocktail and bottom section to 1% cocktail. Mean rates are in nl·min^{-1}·gl^{-1}. The Δ rate is (on-off)/off rate (%); Δ log volume is difference in mean log transformed final volumes after 30 min cocktail stimulation on drug off drug. Significance is two-tailed t-test of log transformed data for glands present in all tests. $P \leq 0.001$ was considered significant. For test consistency, see text and Supplementary Figure 2.

Supplementary Table 2: M-sweat rates off and on ivacaftor.

	Exp #	Subj	GT	Sex	Site	n total SGs	SGs all tests	Mean Rate OFF (± SD)	Mean Rate ON (± SD)	Δ rate (%)	Δ log vol (%)	significance
1	**Ex1**	**S1**	Hz	M	L Tr	63	42	2.50 ± 0.21	2.64 ± 0.18	6%	5%	0.150
2	**Ex1**	**S1**	Hz	M	R Pk	54	44	1.95 ± 0.53	1.95 ± 0.32	0%	0%	0.982
3	**Ex1**	**S2**	WT	F	L Tr	112	70	1.87 ± 0.36	2.13 ± 0.05	14%	9%	0.006
4	**Ex1**	**S2**	WT	F	R Pk	90	52	1.86 ± 0.86	1.98 ± 0.22	6%	1%	0.852
5	**Ex2**	**S4**	WT	M	L	74	41	2.09 ± 0.29	2.34 ± 0.38	12%	9%	0.015
6	**Ex2**	**S4**	WT	M	R	43	31	1.62 ± 0.55	2.11 ± 0.09	31%	16%	0.0005
7	**Ex2**	**S5**	WT	F	L	57	27	1.74 ± 0.11	1.41 ± 0.34	-19%	-13%	0.0009
8	**Ex2**	**S5**	WT	F	R	48	32	0.85 ± 0.37	0.58 ± 0.26	-31%	-18%	1.54E-08
10	**Ex3**	**S1**	Hz	M	L	71	42	2.72 ± 0.12	2.93 ± 0.38	8%	-5%	0.032
11	**Ex3**	**S1**	Hz	M	R	59	44	1.14 ± 0.13	1.08 ± 0.05	-5%	3%	0.299
12	**Ex3**	**S3**	Hz	F	L	81	44	1.50 ± 0.11	1.55 ± 0.12	3%	5%	0.037
9	**Ex2**	**S3**	Hz	F	L	95	55	1.47 ± 0.20	1.52 ± 0.07	3%	3%	0.912
13	**Ex3**	**S6**	Hz	F	L	73	27	2.99 ± 0.73	3.06 ± 0.14	2%	3%	0.135
14	**Ex3**	**S6**	Hz	F	R	89	31	3.25 ± 0.50	2.67 ± 0.42	-18%	-8%	0.014
15	**Ex3**	**S7**	WT	F	L	93	56	1.95 ± 0.35	2.21 ± 0.23	13%	7%	0.0004
16	**Ex3**	**S7**	WT	F	R	87	53	1.59 ± 0.97	2.00 ± 0.21	26%	26%	1.02E-17
16*	**Ex3**	**S7**	WT	F	R*	87	53	2.10 ± 0.52	2.00 ± 0.21	-2%	-3%	0.008
17	**Ex3**	**S8**	WT	M	L	87	30	4.08 ± 0.24	3.89 ± 0.51	-5%	-4%	0.118
18	**Ex3**	**S8**	WT	M	R	82	45	3.28 ± 0.41	3.68 ± 0.46	12%	4%	0.016
	Combined data from both arms											
	**Ex1**	**S1**	Hz	M	L+R		86				2%	0.290
	**Ex3**	**S1**	Hz	M	L+R		91				-1%	0.772
	**Ex1**	**S2**	WT	F	L+R		122				5%	0.030
	**Ex2**	**S4**	WT	M	L+R		72				12%	0.033
	**Ex2**	**S5**	WT	F	L+R		59				-16%	2.29E-10
	**Ex3**	**S6**	Hz	F	L+R		58				-3%	0.190
	**Ex3**	**S7**	WT	F	L+R		109				12%	5.37E-16
	**Ex3**	**S8**	WT	M	L+R		75				1%	0.574
	Means						83.7				2%	

P values based on paired t-tests of log-transformed data. Top panel: responses by subject/arm; bottom panel combined data. Columns as described for Supplementary Table 1 except Δ log volume is mean log transformed volumes after 10 min methacholine stimulation. Correlations for test consistency were not run on M-sweat data.

Supplementary Table 3. Estimates of random effects derived from Linear Mixed Models analyses

**Subject**	**Conc (%)**	sd(Int)	**Gland** sd(Slope)	cor(I, S)	**Week** sd(Week)	**Error** sd(Error)
S1	**1**	0.290	0.150	-0.37	0.0523	0.252
S1	**100**	0.267	0.105	0.07	0.000	0.280
S2	**100**	0.233	0.071	-0.65	0.110	0.320
S3	**1**	0.383	0.147	-0.69	0.040	0.208
S3	**100**	0.343	0.105	-0.67	0.021	0.187
S4	**100**	0.190	0.112	0.38	0.077	0.286
S5	**100**	0.301	0.114	-0.65	0.127	0.269
S6	**1**	0.237	0.063	-0.37	0.055	0.202
S7	**1**	0.200	0.071	-1.00	0.081	0.227
S8	**1**	0.255	0.164	-0.82	0.089	0.228
Average	**1**	0.273	0.120	-0.65	0.063	0.223
Average	**100**	0.267	0.101	-0.30	0.067	0.268
Pooled	**1**	0.260	0.118	-0.57	0.058	0.228
Pooled	**100**	0.273	0.101	-0.28	0.074	0.277

Estimates of the random effects, i.e., standard deviations and correlations, derived from Linear Mixed Models analyses of sweat secretion rate produced by ivacaftor, arrayed by Subject and by level of concentration of the β-adrenergic cocktail. Estimates were obtained using the models given in Eqs. (1)-(3) in the "Linear Mixed Models Analysis" section of the Electronic Supplementary material. Analyses of the pooled data across Subjects also yield standard deviations (not shown in Supplementary Table 3) for the Subject random effect of 0.238 and 0.180 when Conc = 1% and Full, respectively.

REFERENCES

1 Wine, J. J. *et al.* In Vivo Readout of CFTR Function: Ratiometric Measurement of CFTR-Dependent Secretion by Individual, Identifiable Human Sweat Glands. *PLoS ONE* **8**, e77114, doi:10.1371/journal.pone.0077114 PONE-D-13-25271 [pii] (2013).

2 Char, J. E. *et al.* A Little CFTR Goes a Long Way: CFTR-Dependent Sweat Secretion from G551D and R117H-5T Cystic Fibrosis Subjects Taking Ivacaftor. *PLoS ONE* **9**, e88564, doi:10.1371/journal.pone.0088564 PONE-D-13-49924 [pii] (2014).

3 Bates, D., Mächler, M., Bolker, B. & Walker, S. Fitting Linear Mixed-Effects Models Using lme4. *Jounal of Statistical Software* **67**, 1-48, doi:10.18637/jss.v067.i01 (2015).

4 R: A language and environment for statistical computing. R Foundation for Statistical Computing (Vienna, Austria. URL, 2017).
